# Depth-Resolved FTIR-ATR Imaging Studies of Coating Degradation during Accelerated and Natural Weathering—Influence of Biobased Reactive Diluents in Polyester Melamine Coil Coating

**DOI:** 10.1021/acsomega.2c02523

**Published:** 2022-06-30

**Authors:** Alexander Wärnheim, Camilla Edvinsson, Per-Erik Sundell, Golrokh Heydari, Tomas Deltin, Dan Persson

**Affiliations:** † Division of Materials and Production, Department of Corrosion, Research Institutes of Sweden, Isafjordsgatan 28A, Kista 16407, Sweden; ‡ School of Engineering Sciences in Chemistry, Biotechnology and Health, Department of Chemistry, Division of Surface and Corrosion Science, KTH Royal Institute of Technology, Stockholm 10044, Sweden; § SSAB Europe, SDEDN 93, Borlänge 78184, Sweden; ∥ Nordic United Coatings, Cindersgatan 16, Helsingborg 25225, Sweden

## Abstract

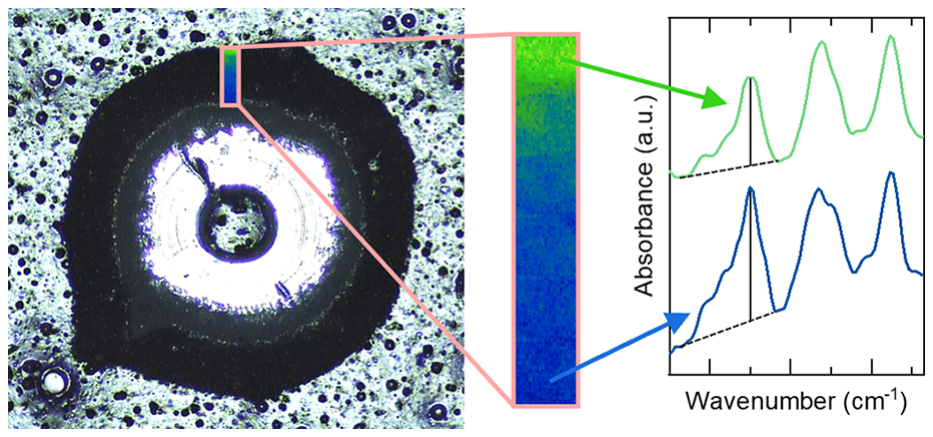

Improved methods to assess the degradation of coil coatings to approximate lifetime have been an area of academic and industrial interest for decades. This work aims to elucidate the differences in the degradation behavior of two coil coating systems: one standard commercial formulation and one formulation with a significant addition of biorenewable reactive diluents. Depth-resolved degradation behavior of samples exposed to accelerated and natural field weathering is assessed. Focal plane array attenuated total reflection-Fourier transform infrared spectroscopy was used to acquire high-resolution chemical data from a sloping cross section. The results agreed with conventional photoacoustic spectroscopy. Degradation profiles for the two coatings were significantly different, with the biobased samples showing a more durable behavior. This study provides a method for detailed assessment of coating degradation, giving a good estimation of its durability. This is both a way to compare the performance of coating systems and to improve the understanding of the impact of exposure conditions, paving the way for the development of more sustainable coil coatings.

## 1 Introduction

Coil coatings are highly durable organic coatings applied onto metal substrates to improve their appearance and to protect the substrate material from corrosion. There are many types of coil coatings optimized for various applications. One of the most widely used systems consists of thermosetting polyester resins with a melamine cross linker. In line with current needs of a sustainable society, recent years have seen significant efforts to produce coil coatings of high quality with a reduced environmental impact. For instance, coatings with reactive biorenewable diluents (BRDs) have been introduced to the market with the aim to replace fossil-based volatile diluents.^1,2^

The long-term durability is a critical property of a coil coating since the thin organic protective layer must withstand very harsh conditions in outdoor applications. The two most important factors that affect the long-term durability of the coating are photooxidation initiated by ultraviolet light and/or hydrolysis caused by humidity.^3−5^ This means that a cold and wet coastal climate can affect a coating in a very different way as compared to a hot and humid subtropical climate. In addition, other factors such as temperature variations, pollution, and chemical species (e.g., ions) are factors that also have an impact on the durability in different ways.^6−8^ The multifaceted nature of the degradation processes makes realistic approximation of coating service life in different climates complicated and an area continually in focus.^7,9,10^ Further, there is currently a trend for the replacement of well-known fossil-based chemical components that have been in use for decades with biobased chemicals which may change the degradation behavior as well as the coating durability. This makes it even more important to be able to assess and understand the degradation behavior on a deeper level. Deeper understanding allows for more accurate lifetime predictions, which is better in terms of risk management.

Coil coating manufactures have long relied on outdoor exposure and appearance-based measurements, such as gloss retention and color change to monitor the condition of the coating.^11,12^ A major limitation of these methods is the long period of time (years) required for significant changes to take place. Although accelerated weathering tests have been developed, results obtained from such methods can be, dependent on the environment, difficult to correlate with long time outdoor exposures.^7,10,13^ In addition, comparisons of chemical degradation effects on specific systems, such as polyester melamine, after field testing and accelerated weathering are rare. One example of such a study is that of Zhang et al., which reported on coatings with different pigments exposed in accelerated weathering (QUVA) and after weathering exposures at a field site in China.^14^ The results showed among other things that different degradation mechanisms related to the dissolution of pigmentation in acid rain occurred for the outdoor weathering exposures as compared to that for the accelerated QUVA weathering. In addition, degradation at the surface level of the coating did not necessarily give a valid indication of the state of the bulk material.

To choose between available, or even develop new, accelerated testing methods, it is important to understand which environmental factors cause degradation in coil coatings and how to quantify different degradation modes. Careful analysis of the local environment and degradation data from outdoor exposures needs to be compared with accelerated testing methods for accurate assessments to be made. In recent years, several studies that focused on utilizing multiple techniques to evaluate changes in coating chemistry also at an early stage of exposure have been published.^15−18^

A sensitive spectroscopic technique, such as Fourier transform infrared (FTIR), has the advantage of allowing the early stages of degradation to be detected and therefore reduces the time required for assessments.^19−21^ Different experimental approaches can be employed to acquire infrared spectra of organic coatings. One of the most common methods is FTIR-ATR (attenuated total reflection), which is relatively fast and simple. However, this method normally only collects data from the surface of the coating of the material. Although the information depth may be tailored to some degree by variations in incidence angle of the infrared light^22^ or by using different internal reflection elements,^23^ the information depth in FTIR-ATR measurements rarely exceeds 2 μm.^24^ This is due to physical limitations imposed by the wavelengths of the incoming light, refractive index of the samples, and available numerical apertures. To overcome this limitation and acquire depth-resolved information while still using FTIR-ATR, Mallégol et al. used micro-lapping. This is a destructive method where the samples are slowly and carefully ground down in 3 μm increments, and successive FTIR-ATR measurements between each grinding session are performed.^25^ Another alternative to acquire depth-resolved chemical information is infrared photoacoustic spectroscopy (PAS). It is a non-destructive technique that can be employed in continuous or by a step-scan scanning mode to provide chemical information from different depths by varying the modulation frequency. PAS has been used by several people to analyze coil coating systems.^8,26−28^ It does, however, have a drawback in that information on the thermal diffusivity of the material is needed to accurately estimate the penetration depth of the thermal vibrations. Furthermore, data is collected from the entire sampling depth across a relatively large surface area, making local defects very challenging to spot. In addition, saturation effects in the spectra will occur at a depth corresponding to the optical absorption depth, which affect relative infrared band intensities and complicate interpretation of depth-resolved measurements.^24^

By using a focal plane array (FPA) detector, it is possible to make a large number of simultaneous ATR measurements with a lateral resolution of 3–4 μm.^29^ In a previous work, Persson et al. used FPA FTIR-ATR imaging over a conical drill hole to provide detailed depth-resolved information about coating degradation.^30^

This project further develops the technique by improving the data analysis, verifying results with complimentary measurements, and comparing the results of different coil coating systems. Complementary depth-resolved information about the degradation was also provided by FTIR-PAS. The overall focus is to elucidate the similarity and difference in degradation phenomena of two coatings under correlative exposure conditions: one standard commercial polyester coil coating and one coating with a substantial amount of fossil solvents replaced by biorenewable reactive diluents. The study includes depth-resolved studies of the degradation of black coatings after accelerated weathering in QUVA and the same systems exposed for natural weathering at two field sites. More informative characterization methods provide a better understanding of the degradation behavior and a possibility to develop accelerated testing methods for coatings with biobased components.

## 2 Experimental Section

### 2.1 Materials

Coil coating formulations were based on commercial OH-functional polyester resins, crosslinked with hexa-methoxy methyl melamine (HMMM) in a ratio of 85:15. The formulations contained carbon black pigmentation, structuring agents, UV-stabilizers, and solvents. Both systems were applied onto polyester melamine primed, hot-dipped galvanized steel sheets with a Ti-based pretreatment.

The samples denoted “standard” were applied in an industrial production line and the analyzed topcoat had a dry thickness of 20 μm.

The coatings denoted “biobased” contained a renewable reactive diluent in the form of rapeseed methyl ester (RME) and were applied using a wire-wound drawdown bar to achieve a topcoat with a dry thickness of 20 μm. The polyester resins contains isophthalic acid, phthalic anhydride, neopentyl glycol, ethylene glycol, and adipic acid. The relative proportion in the resin used for the biobased coating was slightly modified to allow for the transesterification reaction with RME. The films were cured at 360 °C for 30 s to obtain a peak metal temperature of 232–241 °C.

The *T*_g_ of the unexposed coatings as measured by the coating supplier was in the range of 40–45 °C. *T*_g_ was determined by dynamic mechanical analysis (DMA) according to conditions described by Johansson et al.^31^

### 2.2 Exposure Conditions

Accelerated weathering was performed in a QUVA chamber (The Q-Panel Company) equipped with UV-A 340 nm fluorescent tubes (295–400 nm) with a peak irradiance of 0.89 W/m^2^ at 340 nm. The samples were exposed according to the EN13523-10 standard, which consists of 4 h of dry UV-A irradiation at 60 °C, followed by 4 h of condensation humidity at 40 °C. The total exposure time was 2000 h. The field exposures were performed at two different locations. The first one is a marine field station at the Swedish West Coast chosen for its long time of wetness (TOW) and aggressive electrolytes, Bohus-Malmön. Here, TOW is defined according to ISO 9223 and is the period of time where the temperature is above 0 °C and the relative humidity (RH) is above 80%. The samples were mounted on racks at 45° angle facing south and exposed for 3 years. The second exposure site was Atlas’ Florida Benchmarking (henceforth referred to as “Florida”) site located close to Miami approximately 27 km inland from the Atlantic Ocean. The samples were mounted at 45° angle facing south and exposed for 2 years. This site is chosen for high solar UV dosage and high humidity. Additional information about the test sites is provided in Table 1. The TOW for the two field exposure sites was approximated using the yearly average temperature and RH using a method from Tidblad et al.^32^

**Table 1:** Climate Conditions at the Field Exposure Sites

	Atlas’ South Florida Test Service, Miami, Florida^33^	Bohus-Malmön, Kattesand, Swedish West Coast^34^
latitude	25° 52′ N	58° 20′ N
longitude	80° 27′ W	11° 20′ E
elevation (m)	3	40
temperature, yearly average (°C)	26.7	10.4
relative humidity, annual mean	78%	81.5%
annual precipitation (mm)	1685	802
total solar radiant exposure (MJ/m^2^)^a^	6588	3684
distance from sea (km)	27	0.35
calculated time of wetness (%)^b^	57	54

a Radiant exposure measured at a latitude tilt angle (26° south)

b Using the method from Tidblad et al.

### 2.3 FTIR Spectroscopy and FPA Imaging

Both FTIR-ATR and FTIR-ATR FPA measurements were performed using a Bruker Vertex 70 spectrometer with a Hyperion 3000 microscope accessory. The ATR objective had a numerical aperture of 0.6 and an internal reflection element consisting of a germanium crystal. The diameter of the cicular contact area of the crystal is 100 μm and the reflection angle is 45°.

ATR measurements were performed on three different sites on each sample using the spectral region 600–4000 cm^–1^ and a single element MCT detector. Background and sample measurements were collected using 256 scans at a resolution of 8 cm^–1^. FPA imaging was performed as described in ref (30). The spectral region 870–3850 cm^–1^ was measured using a multi-element FPA detector with a 64 × 64 detector array raster and 2 × 2 binning, resulting in a 32 × 32 pixel spectral array at each measurement site. The field of view was 33.8 × 33.5 μm for the FPA measurements. Both background and sample measurements were taken using 500 scans. Before FPA measurements were performed, a manually spun commercially available conical low-angle Säberg coating drill was used to provide access to a cross section in the coating.

### 2.4 Photoacoustic Spectroscopy (PAS) FTIR

An MTEC Model 300 photoacoustic cell was used to perform continuous rapid scan photoacoustic spectroscopic (PAS) FTIR measurements. Four modulation frequencies, 2.5, 5, 10, and 20 kHz, were used with 512, 1024, 2048, and 4096 scans, respectively, to achieve a comparable signal-to-noise ratio. The sample chamber was purged with helium for at least 20 s using a flow rate of approximately 15 cm^3^/s before each measurement. A small amount of magnesium perchlorate (Mg(ClO_4_)_2_) was placed in the sample chamber and used as a desiccant. All measurements were taken at 8 cm^–1^ resolution.

### 2.5 Gloss Retention

Three gloss measurements were performed on each sample using an Elcometer 408. The measurements were conducted at 60° as per the standard for semi-gloss surfaces.

## 3 Results and Discussion

### 3.1 Surface Analysis

FTIR spectra of polyester melamine coatings show many characteristic bands which can be used for evaluation of the degradation.^22,35^ It should be noted that the spectra are normalized to the absorbance of the 1375 cm^–1^ peak, which remains relatively stable throughout the weathering.^30,36^

**Figure 1:**
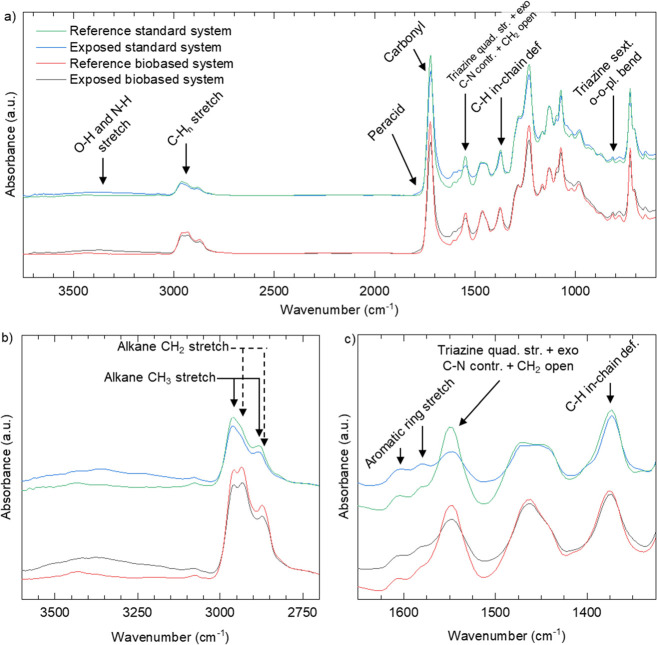
FTIR-ATR spectra of the standard (top) and biobased (bottom) coating samples of unexposed reference samples and degraded samples exposed in Florida. Each spectrum is taken from a single site. (a) Overview of the spectra, (b) detailed spectra in the region 3750 to 2500 cm^–1^, and (c) detailed spectra in the region 1650 to 1325 cm^–1^.

Figure 1 shows representative FTIR-ATR spectra of standard and biobased samples before and after weathering in Florida. Reference (unexposed) panels show bands at 1550 cm^–1^, assigned to the quadrant stretching of the triazine ring and contraction of C–N attached to the ring, coupled with CH_2_ and CH_3_ bending vibrations, and the latter is attributed to the triazine ring sextant out-of-plane bending.^35,37^ In addition, a smaller band at 815 cm^–1^ has been assigned to the triazine sextant out-of-plane bending.^35,37^ Bands attributed to CH_2_ and CH_3_ stretching vibrations are present in the 2800–3000 cm^–1^ region. The distinct carbonyl C=O stretching band at 1735 cm^–1^ and the band attributed to in-chain CH_2_ wagging and CH_3_ bending from 1375 cm^–1^ are present in both systems.^24,35^ Differences between the spectra from the reference and biobased systems include the intensity of the bands associated with CH_2_ stretching and scissoring vibrations at 2930 cm^–1^. This is a clear indication that the long aliphatic carbon chains of RME are chemically incorporated into the cured paint.

Several spectral changes can be seen after weathering. The two most prominent ones include the increased intensity of the O–H and N–H stretching vibrations associated with photooxidation which can be seen in the broad area between 2500 and 3700 cm^–1^, Figure 1b, as well as the broadening and decrease in absorbance for the peak located at 1550 cm^–1^, Figure 1c.^27,37,38^ The latter band is sensitive to changes of the methylol groups attached to the triazine ring, which makes it a convenient way to quantify coating degradation.^27,30,35^ In addition to these changes, the carbonyl peak at 1735 cm^–1^ lowers in intensity and shows a slight broadening due to photooxidation, and a shoulder at 1780 cm^–1^ that is associated with aldehyde and peracid is formed.^5,39^ Changes in the 1550 cm^–1^ band intensity as well as the O–H and N–H bands have all previously been associated with the degradation process of polyester melamine.^25,27,38,40,41^

The degradation shown in the spectra was quantified using melamine substitution functionality loss (MSFL), sometimes denoted melamine degradation, loss of melamine, or triazine substitution index.^14,27,30^
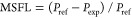
1where *P*_ref_ = (*I*_1550_/*I*_1375_)_ref_ is the absorbance of the 1550 cm^–1^ triazine peak in the reference sample, normalized against the 1375 cm^–1^ peak assigned to the carbon chain backbone of the coating material, and *P*_exp_ is the corresponding normalized peak height in the spectra for the analyzed sample area after exposure.

Data analysis was performed using the software Quasar (v1.1.0).^42,43^

**Figure 2:**
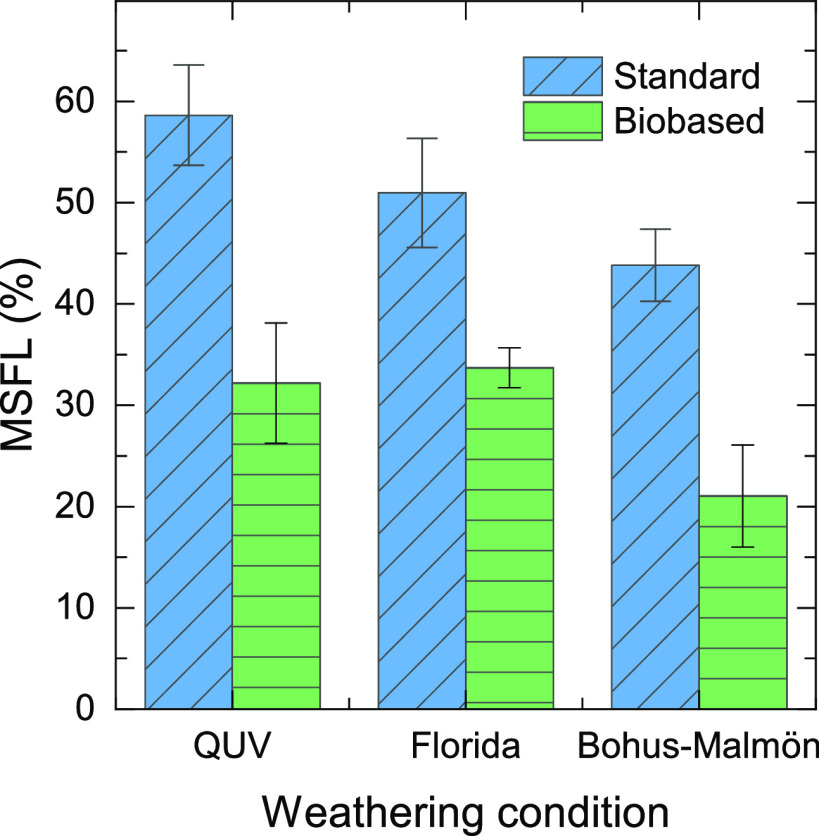
MSFL indices from ATR-FTIR measurements of the standard and biobased coating surfaces after different exposures.

**Figure 3:**
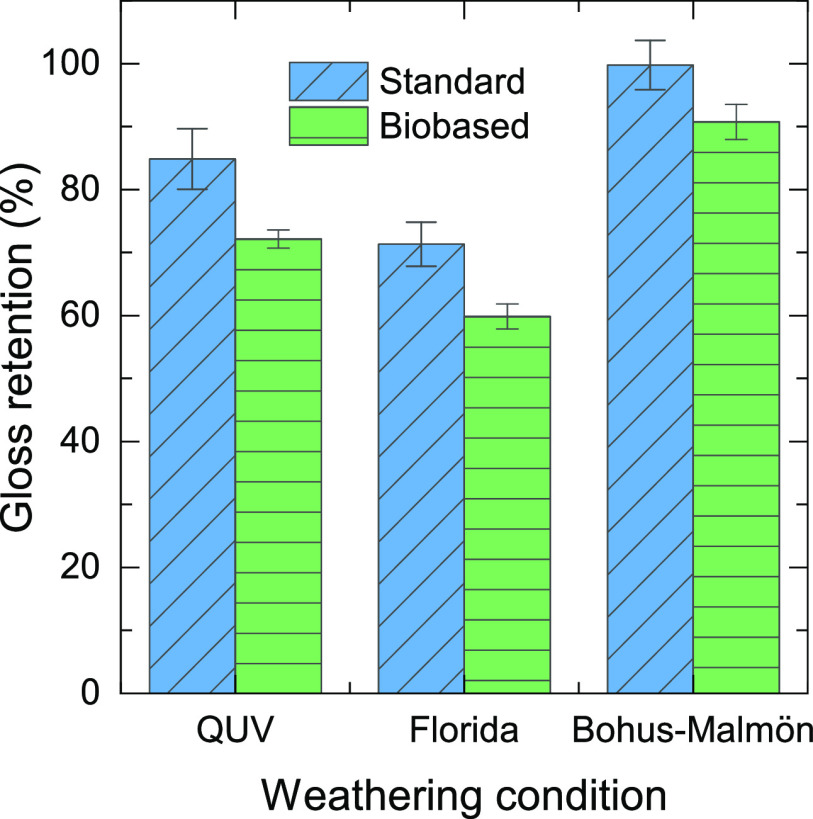
Gloss retention for standard and biobased coating surfaces after different exposures.

MSFL from ATR measurements of the sample surfaces is shown in Figure 2. The biobased samples show lower degradation compared to the standard samples regardless of exposure conditions. Weathering at Bohus-Malmön had the smallest effect on both coatings, despite the fact that the test time was 1 year longer at Bohus-Malmön than in Florida. For the standard samples, accelerated QUV testing showed a larger MSFL as compared to the weathering in Florida. In contrast, the biobased sample showed similar degradation in the QUV test and during exposure in Florida with a difference smaller than the standard deviation. The gloss retention (Figure 3) was lowest after the Florida exposures for both samples, followed by the QUV, and Bohus-Malmön resulted only in a very small change in gloss retention. Note that the biobased samples retained a lower gloss as compared to the standard samples. Previous studies have shown that spectral changes and gloss retention can show poor correlation.^14^

The samples exposed in field sites were subjected to a multitude of factors which may affect the degradation of the coatings. It is generally believed that UV radiation and moisture are the two factors that have the largest impact on the degradation of coil coatings.^4,5,21^ Nguyen et al. have also shown that an increased humidity increases the photolysis rate in a partially methylated melamine acrylic polymer and called this phenomenon moisture-enhanced photolysis (MEP).^41^ For the exposures at the Florida site, total solar radiation is nearly twice the radiation at the Bohus-Malmön site. Thus, accounting for differences in exposure times (2 years in Florida vs 3 years at Bohus-Malmön), the amount of solar radiation received was approximately 30% higher for Florida samples. Although the mean RH was higher in Bohus-Malmön, precipitation was only half that seen in Florida. The TOW was approximately 5% lower for Bohus-Malmön samples. Note that the TOW is based on a standard metal surface and is therefore not necessarily directly translatable to the time the coating is in contact with moisture. Furthermore, the temperature of the Florida site is higher, which will lead to a higher water ingress on the coatings, and also possibly hindering the water egress, especially if temperatures exceed *T*_g_.^11,44,45^ These factors are major contributors to the considerable higher MSFL levels in the samples exposed in Florida as compared to those exposed on Bohus-Malmön.

A direct comparison between the exposure conditions at the field sites and the QUV-accelerated weathering is complicated due to several factors. Gerlock et al. attributed some differences in degradation behavior between these methods to differences in spectral power distribution.^13^ In addition to this, constant high temperatures in QUV, which in this case exceed *T*_g_ of the coatings, have the potential to increase the water ingress and thus MEP to an extent not seen in field exposures.

### 3.2 In-Depth Analysis

One of the most important aspects of coating performance is the durability as a function of depth, which has a critical effect on the service life. The ability to realistically approximate the durability of a coating system is very important. The more accurate the prediction, the better it is in terms of risk management. As investigated in this work, knowledge of the degradation as a function of depth may be achieved by utilizing FPA imaging and PAS. Both methods are sensitive and have the advantage of allowing the early stages of deterioration to be detected and to reduce the time required for assessment.

**Figure 4:**
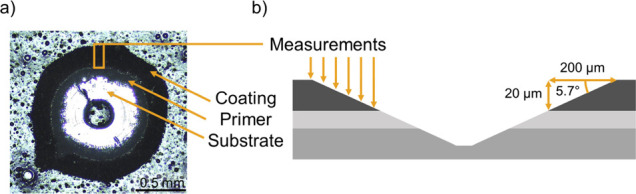
(a) Top view of a conical drill hole from the high angle coating drill acquired using an optical microscope. (b) Schematic (not to scale) of the conical hole showing the positions of the six successive FPA measurements used to measure the cross section.

FPA measurements were performed along drilled-open conical cross sections. No change in temperature before and after drilling could be observed, as measured with an infrared thermometer (Fluke 65). Optical images obtained by the Hyperion FTIR-microscope confirmed that no smearing occurred. Each cross section covered a horizontal area of approximately 34 × 200 μm as indicated by the orange rectangle in Figure 4 a. A side-view sketch of the same area is shown in Figure 4b, and the six arrows show how the difference in measurement location along the surface translates into distances from the original coating surface/air interface. This translation was done using eq 2.
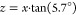
2where *z* is the vertical distance to the surface–air interface, or depth, *x* is the horizontal distance from the edge of the drill hole, and 5.7° is the angle between the substrate and the conical drill hole, as shown in Figure 4b.

**Figure 5:**
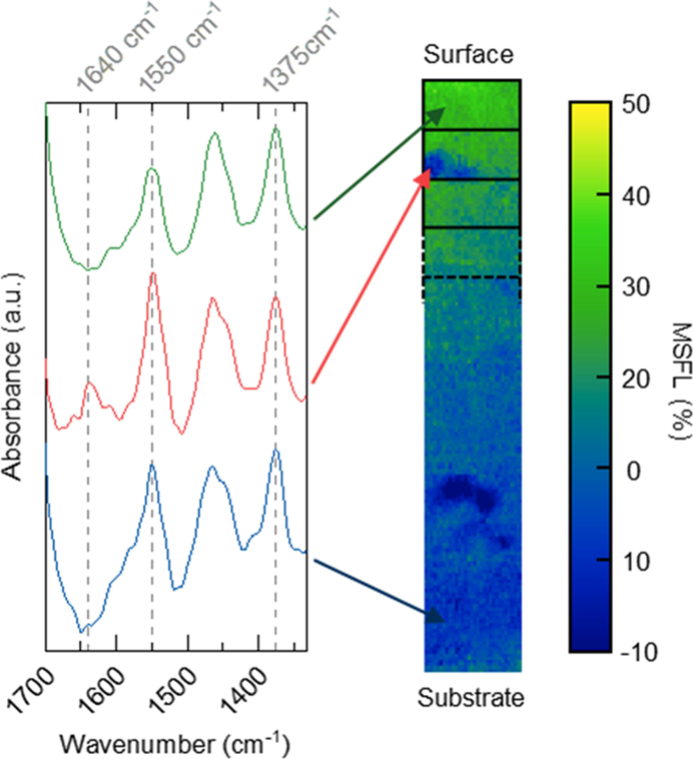
MSFL values from chemical mapping performed on the biobased sample weathered in Florida. Spectra from three different locations with arrows indicating the corresponding pixel in the cross section from which they are collected.

For each collected spectrum, a MSFL value was calculated according to eq 1 and placed at a *z* coordinate (represented as “distance to the surface/air interface”) according to eq 2. Figure 5 shows the cross-sectional image of the biobased sample exposed in Florida and the corresponding spectra in the region 1700–1300 cm^–1^ for three pixels. The spectrum collected close to the substrate, displayed at the bottom of Figure 5, shows a band with high absorbance at 1550 cm^–1^ and thus a MSFL value close to zero. The top spectrum, close to the coating surface, shows a more flattened band at 1550 cm^–1^, with a lower absorbance and thus a higher MSFL. Note that the two dark blue areas, connected to the red arrow, and approximately 1/3 of the way up from the substrate, are not indicative of low chemical degradation. Instead, this is due to polyamide particles, as deduced from the peaks at 1640 cm^–1^ (amide I) and 1540 cm^–1^ (amide II).^37,46,47^ The artificially low MSFL is caused by the way that the band at 1550 cm^–1^ is defined. The peak intensity is defined using a baseline between the absorbance at 1511 and 1623 cm^–1^ and the distance between this baseline and the highest peak in the area. The two amide peaks interfere with both the baseline and the height of the band at 1550 cm^–1^. In practice, this leads to a significantly lowered MSFL at polyamide particles. These areas are not considered when discussing the degradation profiles.

**Figure 6:**
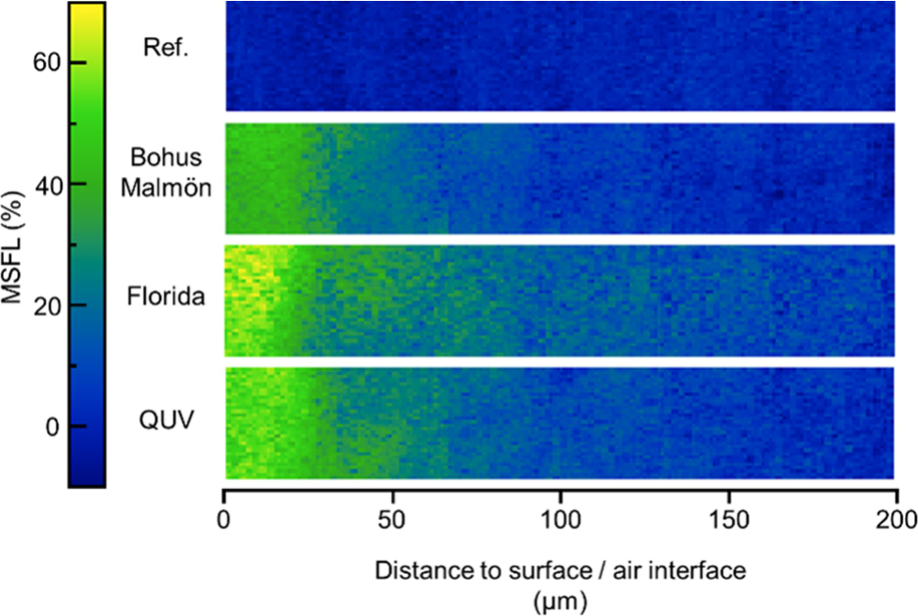
MSFL values obtained by FTIR-ATR FPA imaging in drilled holes for the standard polyester melamine coating. From top to bottom, the images show the unexposed reference sample, samples weathered at Bohus-Malmön in Florida, and samples exposed to accelerated QUV weathering.

Figure 6 shows cross sections of the standard samples exposed in different weathering conditions. The areas close to the surface/air interfaces, furthest to the left in the figure, show that relatively high degradation was caused by outdoor weathering in Florida and accelerated QUV weathering, whereas the weathering at the Bohus-Malmön shows lower values. The degree of degradation in the surface layer is consistent with the results from the ATR measurements in Figure 2. Going deeper into the coatings, MFSL values rapidly decline down to approximately 8 μm in depth after which they remain relatively constant. These results are similar to those obtained by micro-lapping and successive FTIR-ATR measurements on polyester melamine exposed to accelerated weathering by Mallégol et al.^25^ Although the samples weathered using QUV and in Florida have similar MSFL at the surfaces, the Florida samples show higher degradation further into the samples. This could have several possible causes. The most likely is that field exposures caused severe degradation, resulting in damage to the outer parts of the surface that allowed UV light and moisture to penetrate the bulk of the coating more easily.

The reference sample was homogeneous throughout the cross section, and the average peak height of the entire depth was used to calculate *P*_ref_.

**Figure 7:**
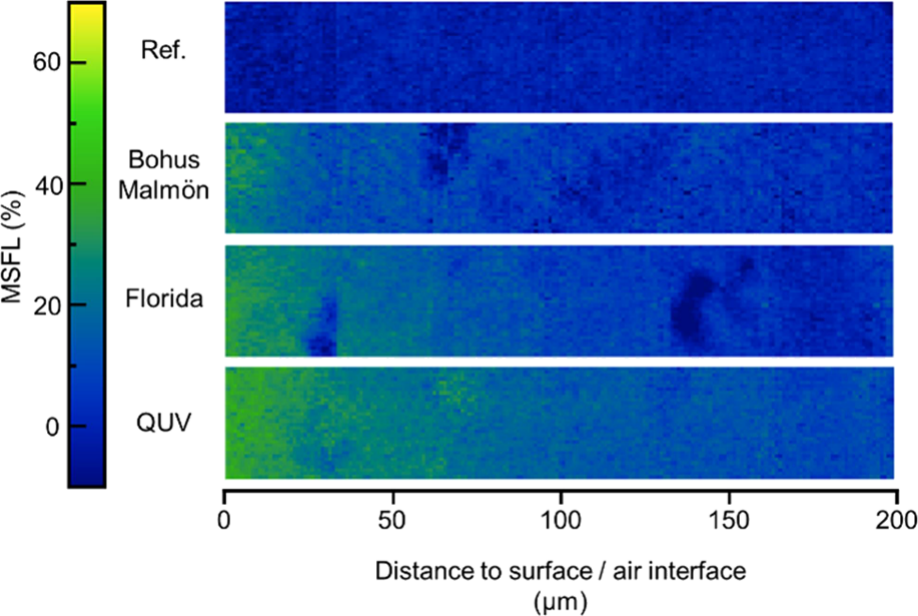
MSFL values obtained by FTIR-ATR FPA imaging in drilled holes for the biobased polyester melamine coating. From top to bottom, the images show the unexposed reference sample, samples weathered at Bohus-Malmön in Florida, and samples exposed to accelerated QUV weathering.

Figure 7 shows cross sections of the biobased samples. The degradation at the surfaces of these samples also correlates well with the ATR measurements in Figure 2. QUV-weathered samples showed the most degradation, followed by Florida and Bohus-Malmön. The degradation profiles in the biobased samples do not show the same sharp decrease in MSFL close to the surface, followed by a flatter slope further into the sample as seen in the standard samples. Instead, the profile shows a relatively flat slope through the entire profile. Note that the dark blue areas in the samples exposed in Florida and at Bohus-Malmön, showing significantly lower MSFL values as compared to the areas around them, are artifacts from the polyamide particles as presented in Figure 5.

**Figure 8:**
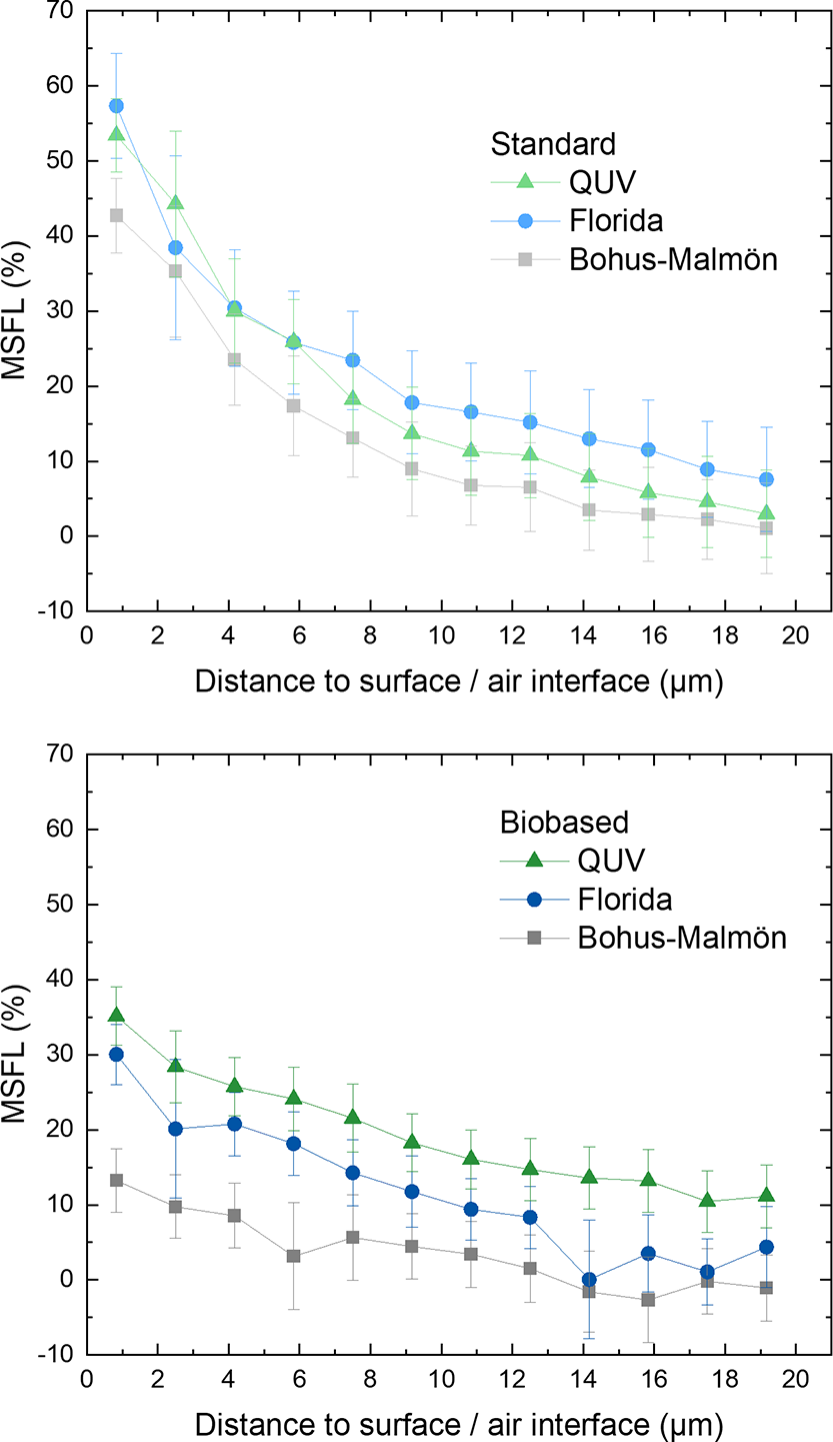
MSFL values obtained by FTIR-ATR FPA imaging in drilled holes at different locations in the cross section. Results from the standard system are on the top and biobased are on the bottom.

To show the difference in the degradation profiles described above, data from Figures 6 and 7 are presented in a scatter plot form in Figure 8. Each point and the connected error bars represent the mean MSFL and the standard deviation within a 16 × 32 pixel area, indicated by the black boxes in the cross section in Figure 5. Note that the sudden drops in the degradation profile at 2 and 14 μm from the surface/air interface in the biobased sample exposed in Florida coincide with the polyamide particles presented in Figures 5 and 7.

The slopes of the MSFL profiles for the different weathering conditions look very similar within the same sample type (i.e., standard and biobased) for all exposure types. However, it should be noted that MSFL is a measurement of the effect of both photolysis and hydrolysis. Further investigations are therefore necessary to understand the mechanisms behind the degradation in detail.

In addition to the FPA imaging, MSFL was also determined using PAS at four different modulation frequencies, 2.5, 5, 10, and 20 kHz. In a system where the photoacoustic signal is limited by thermal diffusion rather than the optical absorption depth, the sampling depth μ_s_ is given by eq 3.^28,48,49^
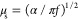
3

with α being the thermal diffusion coefficient and *f* being the optical modulation frequency. The optical modulation frequency is defined as *f* = *n*·*V*_OPD_, where *n* is the wavenumber and *V*_OPD_ is the optical path difference velocity. A thermal diffusivity (α) for a typical polyester resin (1.13 × 10^–3^ cm^2^ s^–1^^50^) was used for calculating the sampling depth in the coating. The modulation frequencies used to perform the in-depth PAS measurement and the corresponding sampling depth are shown in Table 2. A separate *P*_ref_ was calculated for each modulation frequency.

**Table 2:** Approximate Thermal Sampling Depth (Calculated Using *a* = 1.13 × 10^–3^ cm^2^/s)

modulation frequency (kHz)	*V*_OPD_ (cm/s)	calculated sampling depth (μm) [1550 cm^–1^]	calculated sampling depth (μm) [1375 cm^–1^]
2.5	0.16	12.2	12.9
5	0.32	8.6	9.1
10	0.64	6.1	6.5
20	1.28	4.3	4.6

**Figure 9:**
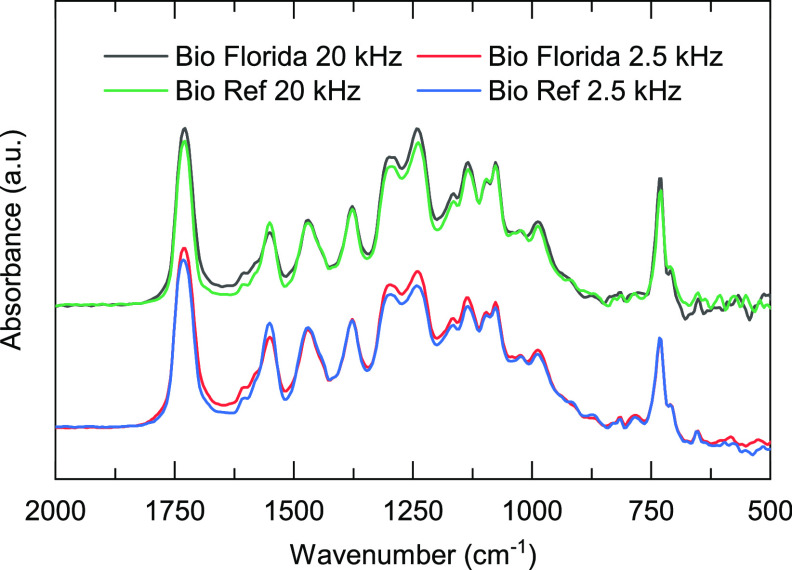
FTIR-PAS spectra of the reference and Florida-exposed biobased polyester melamine coating performed at a modulation frequency of 20 kHz (top) and 2.5 kHz (bottom).

Spectra from measurements performed at 20 and 2.5 kHz on the biobased reference samples and samples weathered in Florida are shown in Figure 9. In comparison to the more surface-sensitive FTIR-ATR measurements in Figure 1, the lowering in absorbance of the band at 1550 cm^–1^ as well as the other signs of degradation mentioned earlier are not much severe for these measurements. In addition, the difference between the spectrum collected using modulation frequencies of 20 and 2.5 kHz, with a sampling depth of 4.3 and 12.9 μm, respectively, is not as noticeable as in the ATR FPA spectra collected at the corresponding depths in Figure 5.

**Figure 10:**
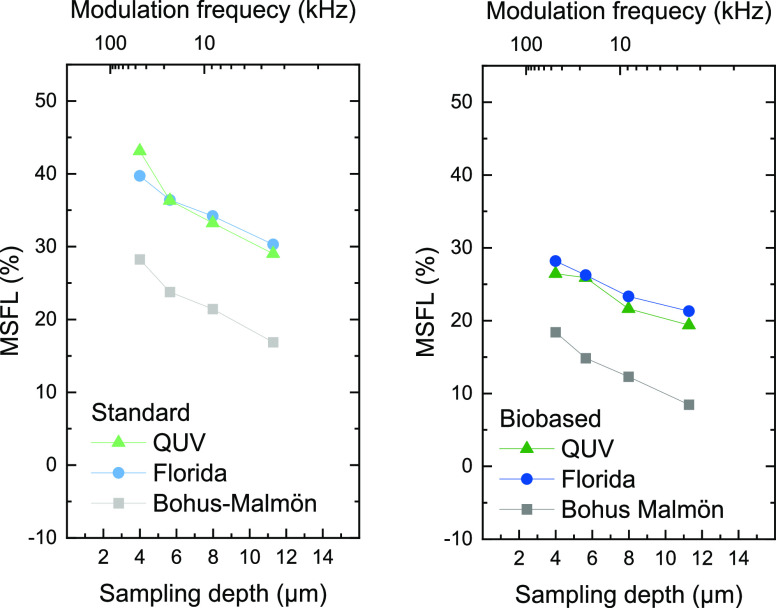
MSFL values obtained from the FTIR-PAS measurements at various modulation frequencies.

Figure 10 shows MFSL values obtained for the different sampling depths. The results were in general consistent with the depth-resolved information provided by the FPA measurements in Figures 6–8. MSFLs are similar for Florida and QUV weathering in both coating types, while samples exposed at Bohus-Malmön show lower degradation for all modulation frequencies. However, PAS measurements show that degradation profiles for the standard samples are less steep and the degree of degradation further into the coating is higher as compared to FPA measurements. This is because PAS results in a spectrum with contributions from the entire volume between the surface and a distance determined by the thermal sampling depth. In contrast, FPA imaging provides local information about the band intensities at a specific location and is limited by the depth of penetration for the IR laser, approximately 0.6 μm with the setup used.^30^

The attenuation of the electric field strength in a material declines exponentially with the depth, which implies a higher contribution from an IR band from the outer parts of the coating for PAS. In Figure 10, the MFSL values are plotted up to a sampling depth of approximately 13 μm as only a small fraction of the light penetrates below this depth. This optical sampling depth was calculated according to an approach by Buffeteau et al.^51^ When this depth is reached, saturation starts to occur, and the optical absorption is the limiting factor which determines the information depth instead of the thermal diffusion. This means that changes in modulation frequency no longer affect the sampling depth and eq 3 is no longer valid.

Despite the principal differences how the PAS and FTIR-ATR FPA imaging measurements are made, the qualitative agreement between the depth-resolved information for both methods is good, considering different weathering conditions and differences between the coating systems. Although a more thorough study is required to understand the mechanistic differences in the degradation behavior of the standard and biobased coatings in detail, it is clear that the introduction of the biorenewable reactive diluent can give rise to chemical differences, which influence the degradation behavior significantly.

## 4 Conclusions

FTIR-ATR FPA spectroscopic imaging was successfully employed to obtain depth-resolved information to compare a standard coil coating to a similar system with an addition of a biobased diluent. The results show that changes in melamine substitution (MFSL), caused by UV and moisture-induced degradation (MEP) as well as inhomogeneities in the form of amide particles, could be imaged with a micrometer-scale resolution. These results agree qualitatively with complimentary infrared PAS measurements, even though the different nature of the measurements makes quantitative comparisons complicated.

Although the in-depth degradation profiles obtained after field exposures and accelerated weathering were similar, it was not proven that the degradation mechanisms are identical for the two cases. Highest degradation was observed for samples exposed 2 years in Southern Florida and QUV-accelerated weathering followed by 3 years’ field exposures at the Swedish west coast (Bohus-Malmön). The high loss of melamine crosslinker functionality at the Southern Florida exposure site is consistent with a dominant MEP of melamine side groups and melamine-polyester linkages. MEP is facilitated at the Southern Florida site by high UV radiation intensities, high humidity, precipitation, and temperature, which presumably facilitates high water uptake in the coating.

The depth-resolved measurements showed different degradation propensities for a standard coating and a coating with the added BRD. The coating with a standard formulation shows high degradation in the outer parts of the coating and a rapidly decreasing degree of degradation further into the bulk of the coating. In contrast, the biobased coating showed a comparatively low difference between the degradation at the surface as compared to that in the bulk.

Accurate depth-resolved information about degradation in coatings is important to determine how differences in formulation and exposure conditions affect degradation modes. In addition, more research is required to verify the validity of accelerated testing methods. This is especially relevant as industry and the market are currently pushing for a fast switch from fossil-based raw materials to greener and preferably renewable ones. The technique demonstrated in this article has been shown to be a useful tool with the potential to aid in both of these goals.

## Abbreviations

ATR: attenuated total reflection
BRD: biorenewable diluent
DMA: dynamic mechanical analysis
FPA: focal plane array
FTIR: Fourier transform infrared
HMMM: hexa-methoxy methyl melamine
MEP: moisture-enhanced photolysis
MSFL: melamine substitution functionality loss
PAS: photoacoustic spectroscopy
TOW: time of wetness
OPD: optical phase difference
